# Development of Nanobody-Expressing Nanosomes for Neutralization of Influenza Virus

**DOI:** 10.4014/jmb.2509.09047

**Published:** 2025-11-18

**Authors:** Taehyun Kim, In-Hwan Jang, Sohyeon Shin, Juhyun Kang, Hyo-Joo Ahn, Sungmin Moon, Juhyun Kim, Ji-Hwan Ryu, Kyung-Ah Lee

**Affiliations:** 1 Department of Biomedical Sciences, Yonsei University College of Medicine, Seoul 03722, Republic of Korea; 2 Brain Korea 21 Project, Yonsei University College of Medicine, Seoul 03722, Republic of Korea; 3 Department of Microbiology & Immunology, Seoul National University College of Medicine, Seoul 08826, Republic of Korea; 4 School of Life Sciences, BK21 FOUR KNU Creative BioResearch Group, Kyungpook National University, Daegu 41566, Republic of Korea; 5 Saeloun Bio Inc., Seoul 08826, Republic of Korea; 6 Department of Otorhinolaryngology, Yonsei University College of Medicine, Seoul 03722, Republic of Korea

**Keywords:** Influenza viruses, nanosomes, *Lactiplantibacillus plantarum*, intranasal administration

## Abstract

Influenza viruses remain a persistent threat to both human and animal health, whereas current countermeasures—vaccination and livestock culling—offer only delayed, partial, or economically burdensome protection. Here, we describe the development of a mucosal nanotherapy based on chromosome-free minicells derived from *Lactiplantibacillus plantarum*, engineered to surface-display a broadly reactive anti-hemagglutinin nanobody. These therapeutic agents, termed “neutralizing nanosomes” present nanobody molecules anchored on the minicell surface that retain full binding functionality against a broad spectrum of influenza viruses, including H1N1. Importantly, intranasal administration of neutralizing nanosomes effectively neutralized H1N1 infection *in vivo*, alleviating physiological symptoms and suppressing viral replication in the respiratory tract of a preclinical mouse model. Unlike vaccines, which require weeks to confer protection, our neutralizing nanosomes provide an immediate barrier at the respiratory mucosa—the primary portal of influenza entry—offering a promising adjunct to existing vaccines and antiviral drugs.

## Introduction

Influenza viruses are RNA viruses of the family *Orthomyxoviridae*, classified into types A, B, C, and D: type A infects humans, birds, pigs, and other animals, type B mainly infects humans, while types C and D cause mild or host-restricted infections [1, 2]. Avian influenza viruses (AIV) are subtypes of influenza virus type A originating and circulating in birds. AIV predominantly infect poultry, such as chickens and ducks, and are classified into two main types: low pathogenic AIV, which causes a drop in egg production, and highly pathogenic AIV, which can result in a mortality rate of up to 100% within 48 h post-infection [3, 4]. AIV subtypes are determined by 16 hemagglutinin (HA) and 9 neuraminidase (NA) surface antigens, leading to combinations such as H1N1 and H7N9 [4, 5].

Traditionally, AIV has been considered species-specific, with minimal cross-species transmission. However, emerging evidence shows that AIV strains originating in poultry have begun infecting pigs and humans, underscoring a growing zoonotic threat [6]. As of mid-2025, the WHO reports no new human infections with H5N6, H7N9, or other rare subtypes in the Western Pacific Region, but remains vigilant about sporadic influenza A cases and emphasizes ongoing zoonotic risk due to continued viral circulation in birds [7, 8]. More recently, between January 2024 and June 2025, the United States documented 70 human highly pathogenic H5N1 AIV infections, including one death (CFR ≈ 1.43%); many were linked to exposure to infected dairy cows and poultry [9].

Although vaccines against AIV do exist—primarily inactivated whole-virus vaccines administered via injection—their efficacy is limited. In the EU, one authorized vaccine does not fully block transmission, and vaccination remains injective and labor-intensive, making it impractical for large-scale implementation in livestock [5]. Furthermore, a recent study demonstrated that inactivated H9N2 vaccines fail to prevent viral replication in the upper respiratory tract of chickens, inadvertently promoting viral evolution and enhancing zoonotic potential [10]. In addition, poultry vaccinated against AIV cannot be exported. As a result, most poultry farms manage AIV outbreaks through mass culling of infected flocks and the imposition of strict movement restrictions. Although this approach is intended to contain viral spread, it leads to considerable national and economic losses. In the case of highly pathogenic AIV outbreaks, mass culling remains the primary control measure—an approach that is both costly and devastating. For instance, between 2022 and 2023, avian influenza outbreaks were reported on 75 farms nationwide, causing significant economic damage [11].

Given these limitations of conventional vaccination and control strategies, there is an urgent need for innovative approaches that can provide broad and effective protection against diverse influenza strains [12]. In this context, diverse vaccine platforms have recently been developed to address the extensive antigenic variation of influenza viruses by targeting multiple HA motifs. In addition to these vaccine platforms, nanobody-based therapeutics have emerged as an alternative antiviral strategy, owing to their structural simplicity, stability, and ease of engineering. Nanobodies, also known as single-domain antibodies, are the smallest functional fragments of heavy-chain-only antibodies originally discovered in camelids. Owing to their small size, high stability, and ability to access hidden epitopes that conventional antibodies may not reach, nanobodies represent a versatile tool for antiviral design [13, 14]. Notably, recent studies have reported the successful generation of nanobody-derived multidomain antibodies with the capacity to recognize a wide range of influenza viruses including AIV, thereby providing a strong foundation for the development of broadly protective influenza vaccines [15].

Building on these advances, the present study aims to develop and evaluate a novel nano-scale neutralizing platform—termed “neutralizing nanosomes”—that can be administered intranasally, thus mimicking the natural route of influenza infection. In this approach, AIV-specific nanobodies were expressed on the cell surface of a probiotic *L. plantarum* and subsequently packaged into minicells [16-18], which are devoid of chromosomal DNA and therefore pose no genetic safety concerns. This design is intended to provide a practical, safe, and cost-effective alternative for large-scale application in livestock populations. By integrating broad-spectrum antiviral activity with the stability and scalability of nanoscale delivery vehicles, neutralizing nanosomes are anticipated to serve as a transformative tool for the rapid, efficient, and affordable prevention of AIV transmission in agricultural settings. Ultimately, this study seeks to establish proof-of-concept for nanobody-based neutralizing nanosomes as a next-generation antiviral platform with potential applications beyond AIV.

## Materials and Methods

### Bacterial Strains and Growth Conditions

*E. coli* was cultured at 37°C with agitation in Luria Bertani (LB) broth. *Lactiplantibacillus plantarum* WJL was grown in de Man, Rogosa and Sharpe (MRS) at 37°C without agitation. When necessary, culture media were supplemented with antibiotics at the following concentrations: kanamycin 50 μg/ml for *E. coli*, erythromycin 200 μg/ml for *E. coli* and erythromycin 10 μg/ml for *L. plantarum* WJL.

### Plasmid Construction and DNA Manipulation

**Fig. 1:**
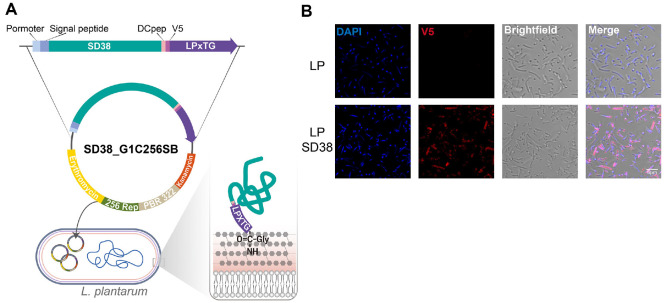
Construction and surface display of recombinant *L. plantarum* expressing SD38. (**A**) Schematic representation of the recombinant expression construct. The SD38 gene was codon-optimized for *L. plantarum* and fused at its C-terminus to a dendritic cell-targeting peptide (DCpep), an V5 epitope tag, and the LPxTG anchoring motif to enable covalent attachment to the bacterial peptidoglycan. The fusion cassette was placed under the control of a constitutive promoter and cloned into the shuttle vector SD38_G1C256SB, containing replication origins and selectable markers for erythromycin and kanamycin resistance. The recombinant plasmid was subsequently introduced into *L. plantarum*, resulting in stable surface expression of SD38 via the LPxTG anchoring system. (**B**) Immunofluorescence staining of recombinant *L. plantarum* strains. Wild-type *L. plantarum* (LP, upper panels) displayed DAPI-stained nucleoids (blue) but no detectable V5 signal (red). In contrast, recombinant strain LP_SD38 (lower panels) showed strong surface-localized V5 fluorescence, indicating successful expression of the nanobody construct. Brightfield images confirm bacterial morphology, and merged panels demonstrate colocalization of V5 signals with bacterial cells. Scale bar, 5 μm.

To enable stable surface display of the broadly neutralizing single-domain antibody SD38, which targets influenza hemagglutinin (HA), in *L. plantarum*, the SD38 coding sequence [15] was codon-optimized according to the codon usage of *L. plantarum*. The synthetic SD38 gene was cloned between the signal peptide and the DC pep-V5-LPxTG cassette of the G1C256SB vector [19] (Fig. 1A). For minicell production, a *L. plantarum* Δ*minD* mutant strain, which was previously reported to efficiently generate minicells without affecting overall cell viability [18, 20]. and in parallel, a recombinant Δ*minD* strain was engineered to simultaneously produce minicells and present nanobody molecules on their surface. First, the minicell-producing Δ*minD* strain was introduced with SD38- G1C256SB plasmid to generate the Δ*minD*::SD38 strain. After selection on MRS media with erythromycin, the strain was verified by sequencing and fluorescence microscopy to confirm successful minicell formation and surface expression of the recombinant proteins.

### Immunofluorescence Staining

*L. plantarum* was cultured overnight in MRS medium. Bacterial cells (10^8^ cells/tube) were harvested by centrifugation at 5,000 ×*g* for 5 min and washed twice with PBS. For fixation, the cell pellet was incubated in 4%paraformaldehyde at room temperature for 10 min, followed by two additional PBS washes to remove excess fixative. The cells were then resuspended in 5% bovine serum albumin (BSA) in PBS and incubated for 1 h to block nonspecific binding. The samples were washed twice with PBS and incubated overnight at 4°C with the primary antibody diluted in 3% BSA in PBS. Following primary antibody incubation, the cells were washed three times for 5 min each with 0.1% Triton X-100 in PBS, then incubated with the secondary antibody and DAPI in 3% BSA in PBS for 20 min at room temperature. The samples were washed three more times for 5 min each with 0.1% Triton X-100 in PBS, followed by two additional washes with 1× PBS for 5 min each. Finally, the samples were mounted onto a confocal dish (SPL; 100350) and analyzed using confocal microscopy (Carl Zeiss; LSM700, Germany). The following antibodies and reagents were used in this study: Primary antibodies: Anti-V5 (1:500; Invitrogen, Catalog # R960-25, USA). Secondary antibodies: Alexa Fluor 568 goat anti-mouse IgG (1:500; Invitrogen, A11004). Fluorescent dye: DAPI (1:1000; Roche, 10236276001, Switzerland) was used for nuclear staining.

### Cell Wall Fraction Preparation

To isolate the cell wall fraction from *L. plantarum*, 10 mL of culture was grown in MRS medium at 37°C until reaching the stationary phase. Bacterial cells were harvested by centrifugation, washed once with PBS, and disrupted using bead beating (6,500 rpm, 3 × 40 sec) with 0.1 mm silica beads. The disrupted cells were centrifuged at 1,000 ×*g* for 1 min, and the resulting supernatant was further centrifuged at 16,000 ×*g* for 30 min at 4°C to pellet the cell wall fraction. The pellet was resuspended in 100 μl of PBS.

### Isolation and Characterization of Minicells

Minicells were isolated from *L. plantarum* WJL Δ*minD*, a minicell-producing strain employed in a previous study [20]. A single colony was inoculated into MRS broth and incubated overnight at 37°C. The overnight culture was diluted 1:1000 into fresh MRS medium (50 ml) and further incubated at 37°C overnight. Cells were harvested by centrifugation at 4,000 ×*g* for 10 min at room temperature to pellet parental cells, and the supernatant was carefully transferred into new conical tubes. Minicells were subsequently pelleted by centrifugation at 7,197 ×*g* for 15 min, resuspended in 500 μl PBS, and filtered through a 0.8 μm syringe filter to remove residual parental cells. The filtered minicells were resuspended in 10 ml fresh MRS medium and incubated at 37°C for 3 h to allow recovery. Ceftriaxone was then added to a final concentration of 100 μg/ml, and the culture was incubated for an additional 4 h at 37°C. Following antibiotic treatment, steps of differential centrifugation and filtration were repeated as described above to further enrich minicells. The final pellet was collected by centrifugation at 11,000 ×*g* for 5 min, washed once with 1 ml PBS, and resuspended. Purified minicells were stored at 4°C until further use. The purity of isolated minicells was routinely assessed by microscopy and chromosome staining to confirm the absence of parental cells. For large-scale preparations (>1 L initial culture volume), the washing volume of PBS was proportionally increased to ensure effective filtration. During syringe filtration, gentle pressure was applied to minimize parental cell contamination, avoiding excessive force and bubble formation. After purification, both LP_SD38 and LP nanosome suspensions were normalized to an approximate concentration of OD_600_ = 2.5. Equal volumes of these standardized samples were subsequently employed in all experimental assays, including animal studies.

### Cell Culture

Madin-Darby canine kidney (MDCK) cells were obtained from American Type Culture Collection (ATCC; CCL-185, USA). MDCK cells were maintained in EMEM (ATCC, 30-2003) supplemented with 10% (v/v) heat-inactivated fetal bovine serum (FBS) (Gibco, 16000-044, USA) and 1% (v/v) penicillin–streptomycin (Gibco, 15140-122). A549 cells were purchased from ATCC (CCL-185) and cultured in DMEM (Gibco, 11995-065) containing 10% (v/v) heat-inactivated FBS, 1% (v/v) penicillin/streptomycin (Gibco, 15140-122). Cells were maintained in 175 cm^2^ flasks (Corning, 431080) at 37°C in 5% CO_2_ incubator. The passage number for all cells used in this study was less than 30.

### Virus Propagation and Plaque Assay

Influenza A virus /Korea/01/2009 (pH1N1), the first pandemic H1N1 influenza A virus (IAV) strain isolated in Korea during the 2009 global outbreak, was propagated in embryonated chicken eggs, which was previously reported [21]. Briefly viruses were harvested via centrifugation of allantoic fluid or culture medium at 1300 ×*g* for 10 min. They were stored at −80°C, and viral titers were determined in a plaque assay. For plaque assay, a monolayer of MDCK cells were inoculated with serially diluted virus for 1 h. After inoculation, the unbound viral particles were washed with PBS, and the cells were overlaid with 1 ml of Minimum Essential Medium (MEM) containing 1 μg/ml of tosyl phenylalanyl chloromethyl ketone (TPCK)-treated trypsin and 1% agarose. After 72 h incubation, the cells were fixed and stained with 1% crystal violet, and viral titers were measured by counting the plaques.

### *In vitro* Virus Infection

A549 cells were seeded 1.8 × 10^5^/well in 12-well plates (Corning, 3513) 24 h prior to infection. On the following day, the H1N1 virus was preincubated with or without the cell wall fraction prepared in serum-free medium for 1 h at room temperature under gentle rocking conditions. After preincubation, cells were washed with PBS and then exposed to one of three conditions: serum-free medium alone (None), virus only (IAV), or the virus–cell wall fraction mixture (LP CWF + IAV or LP_SD38 CWF + IAV), at a multiplicity of infection (MOI) of 1. For infection, viruses were inoculated at 37°C and 5% CO_2_ in a humidified incubator for 1 h. After inoculation, cells were washed with PBS and the medium was replaced with fresh DMEM containing 5% FBS for 24 h.

For assays using nanosomes, IAV particles were pre-incubated with either LP nanosomes or LP_SD38 nanosomes adjusted to an optical density (OD_600_) of approximately 2.5 under the same conditions described above. Following infection and incubation, total RNA was isolated from cells for qRT-PCR analysis of viral gene expression.

### Real-Time Quantitative Polymerase Chain Reaction (qRT-PCR)

Total RNA was isolated from A549 cells and mouse lungs using Hybrid-R (GeneAll Biotechnology, 305-101, Republic of Korea). For lung samples, the superior lobe of the right lung was used. Complementary DNA (cDNA) was synthesized from 500 ng of RNA with random hexamer primers (Invitrogen, N8080127), RNase inhibitor (Applied Biosystems, N8080119, USA), dNTPs (Applied Biosystems, N8080260), and M-MLV reverse transcriptase (Invitrogen, 28025013). For quantitative PCR (qPCR), KAPA SYBR FAST qPCR master mix (2X) (Roche, KK4605) was used according to the manufacturer’s instructions. qRT-PCR was performed using QuantStudio 3 Real-Time PCR System (Thermo Scientific, USA). Gene expression levels were evaluated using the comparative Ct method (2-ΔΔCt method). Primers used for real-time qPCR in this study are listed in following table.


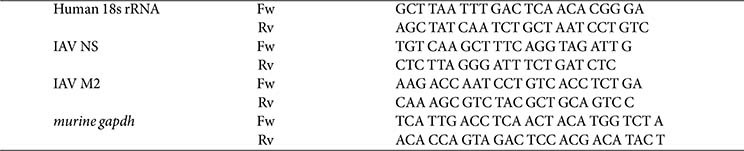


### Mouse Experiments

All mouse experiments were approved by the Institutional Animal Care and Use Committee (IACUC) at Yonsei University College of Medicine (protocol no. 2022-0257) and performed in accordance with the Association for Assessment and Accreditation of Laboratory Animal Care International (AAALAC International, facility no. 001071) guidelines. 4-week-old male C57BL/6 mice were obtained from Orient Bio (Republic of Korea) and housed in Animal Biosafety Level 2 facility with under a 12 h light-dark cycle at 20 ± 2°C, with 50 ± 5% humidity, light intensity of 150–300 lux, ventilation of 10–15 air changes per hour, and noise levels under 60 dB. Mice were randomly assigned into three groups (*n* = 5 per group) and acclimatized for 1 week prior to experimentation.

### *In vivo* Intranasal Administration and Infection

Following acclimatization, mice were anesthetized by intraperitoneal injection (i.p.) of a Zoletil: Rompun: PBS mixture (1:1:8, 100 μl). The nanosome mixture of PBS (25 μl) was administered intranasally according to group assignment. Fifteen minutes later, all groups were intranasally challenged with 1 × 10^4^ PFU IAV in 25 μl PBS. Body weights were monitored daily for 8 days post-infection. Mice were sacrificed at 8 days post-infection, and lung tissues were collected for further analyses, including quantitative PCR (qPCR).

### Histopathological Analysis of Mouse Lungs

For histological analysis, the left lung from each mouse was collected and fixed in 4% paraformaldehyde Solution for 24 h. The tissues were then dehydrated, embedded in paraffin, and sectioned at a thickness of 5 μm. The sections were stained with hematoxylin and eosin (H&E) and examined under a light microscope to evaluate inflammatory cell infiltration and tissue damage.

### Statistical Analysis

All statistical analyses were performed using GraphPad Prism (version X; GraphPad Software, USA). Data are presented as mean ± SEM. For comparisons among multiple groups, one-way analysis of variance (ANOVA) followed by Tukey’s multiple comparisons test was applied. Statistical significance was defined as *p* < 0.05. Levels of significance are indicated in the figures as follows: *p* < 0.05 (*), *p* < 0.01 (**), *p* < 0.001(***) and *p* < 0.0001 (****). Differences that were not statistically significant are denoted as “ns.”

## Results

### Construction of Recombinant *L. plantarum* Expressing SD38 Antigen

To enable stable surface display of the broadly neutralizing single-domain antibody SD38 against influenza hemagglutinin (HA) in *L. plantarum*, the SD38 coding sequence was codon-optimized according to *L. plantarum* codon usage. SD38 is of particular interest because it exhibits broad reactivity against multiple influenza A subtypes, including H1, H2, and H5, as well as selected group 2 viruses such as H3 and H7, thereby representing one of the most versatile nanobodies identified to date for anti-influenza applications [15]. For efficient surface expression, the SD38 gene was fused to an LPxTG motif, enabling covalent anchoring of the nanobody onto the peptidoglycan layer of the bacterial cell wall [19]. The final construct, containing the SD38-LPxTG fusion, was cloned into a constitutive expression plasmid under the control of a strong promoter and introduced into *L. plantarum* by electroporation (Fig. 1A). This recombinant strain specifically presents SD38 at the outer surface, enabling direct viral interaction and reflecting robust engineering for functional display.

### Verification of SD38 Display on the Surface of Recombinant *L. plantarum*

To assess successful expression and surface display of the recombinant nanobody SD38, immunofluorescence microscopy was performed. Wild-type *L. plantarum* (LP) did not exhibit detectable fluorescence with anti-V5 antibodies, indicating the absence of non-specific staining. In contrast, the recombinant *L. plantarum* strain (LP_SD38) exhibited strong fluorescence signals corresponding to SD38-V5 (red), which was localized to the cell surface under non-permeabilizing conditions. DAPI staining confirmed the integrity of bacterial cells, and merged images demonstrated pronounced bright field image and V5 signals (Fig. 1B).

Collectively, these results demonstrate that the recombinant *L. plantarum* strain successfully expresses and displays the SD38 antigen on its surface, validating the effectiveness of our plasmid design and anchoring strategy. This system provides a promising platform for the development of mucosal delivery vehicles against avian influenza virus.

### Generation of Neutralizing Nanosomes from Recombinant *L. plantarum* Minicells

**Fig. 2:**
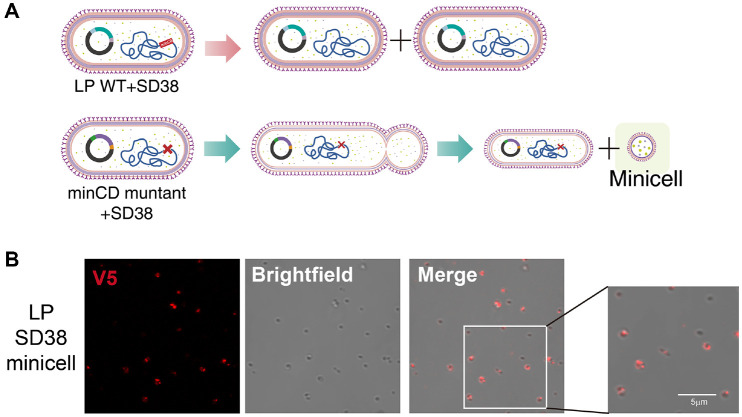
Generation of minicells and surface display of SD38 nanobodies. (**A**) Schematic illustration of minicell formation. Wild-type *L. plantarum* expressing SD38 (LP WT+SD38) undergoes normal cell division, whereas the *minCD* *mutant* expressing SD38 produces anucleate chromosome-free minicells, which can be harvested as nanoscale delivery vehicles. (**B**) Immunofluorescence staining of minicells derived from recombinant strains. Minicells isolated from LP_SD38 strains displayed strong V5-positive signals (red), confirming surface expression of the SD38 nanobody. Brightfield and merged images show clear colocalization of V5 signals with minicell structures, and a magnified inset highlights the surface distribution pattern. Scale bar, 5 μm.

In this study, *L. plantarum*–derived minicells were employed as biologically inert, nano-sized delivery vehicles for nanobody presentation [18, 20]. Because minicells are devoid of chromosomal DNA, they pose no genetic safety concerns while providing a stable and scalable platform for functional protein display, making them particularly suitable for application in livestock populations. To demonstrate the surface expression of nanobodies in minicell-derived particles, recombinant *L. plantarum* strains expressing the SD38 construct were engineered with a mutation in the endogenous *minD* gene [20], thereby promoting minicell formation and facilitating nanosome generation (Fig. 2A). Minicells were subsequently isolated as described and subjected to immunofluorescence staining using an anti-V5 antibody to detect nanobody expression on the surface. Distinct punctate red fluorescence signals were observed on minicells derived from SD38 expressing *L. plantarum*, demonstrating successful display of functional SD38 nanobody molecules on the nanosome the absence of parental cells (Fig. 2B).

The efficient surface presentation of SD38 on these nanosomes suggests that they may directly engage IAV particles. Consequently, subsequent experiments were designed to evaluate the binding affinity of the nanosomes toward IAV and to assess their functional capacity to neutralize viral infection.

### Binding of Nanobody-Expressing Bacteria to influenza virus

To further evaluate whether nanobody molecules displayed on the bacterial surface retained functional binding activity against IAV, cell wall fractions (CWFs) were prepared from either wild-type *L. plantarum* (LP) or recombinant LP_SD38 strains expressing the nanobody constructs.

**Fig. 3:**
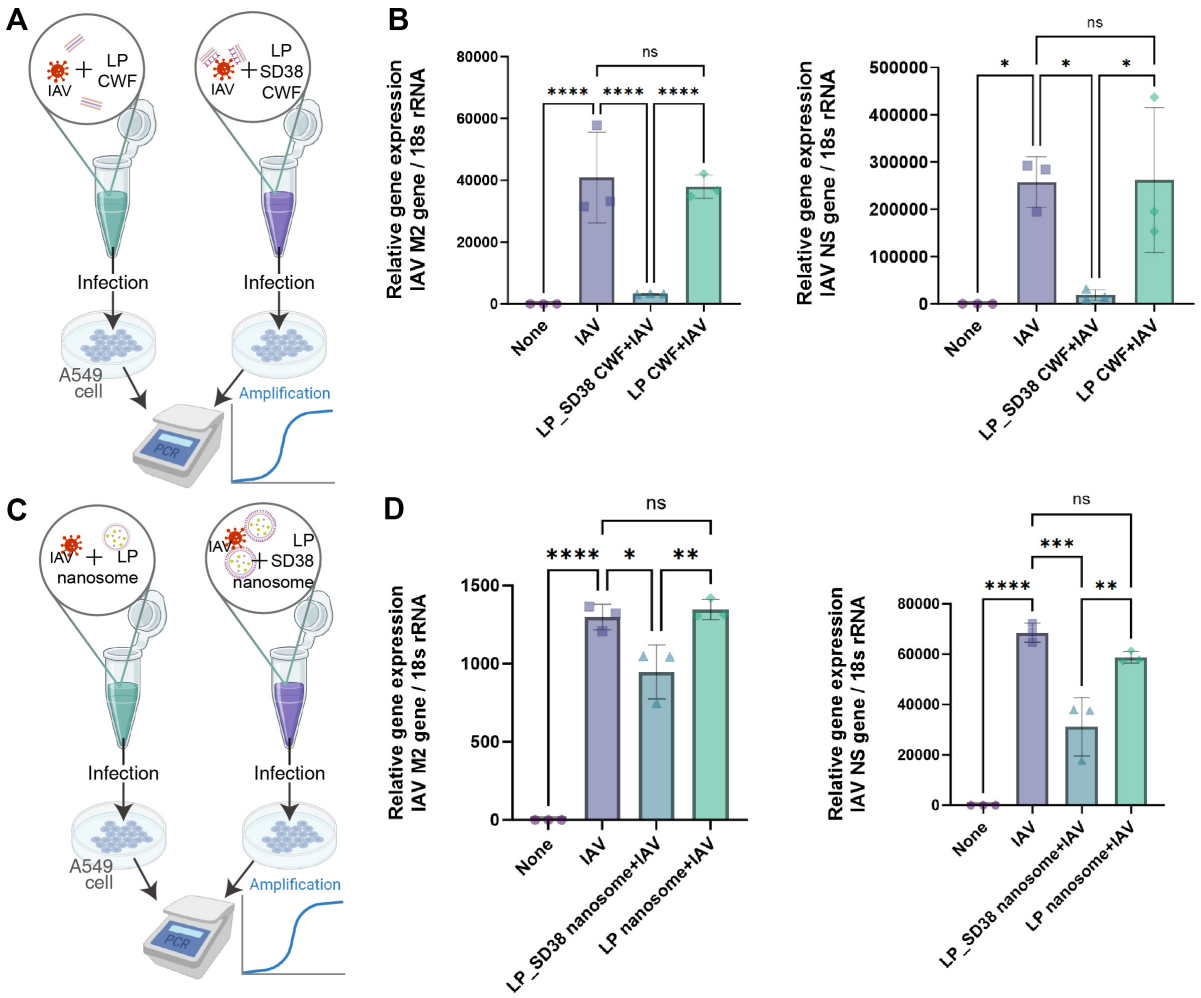
Neutralizing activity of SD38-expressing *L. plantarum* cell wall fractions and nanosome against IAV *in vitro*. (**A**) Schematic representation of the binding assay. Cell wall fractions (CWFs) were prepared from wild-type *L. plantarum* (LP) or recombinant LP_SD38 strains expressing the SD38 nanobody. IAV particles were pre-incubated with each CWF preparation and subsequently used to infect A549 cells. Viral replication was assessed by qRT-PCR analysis of IAVspecific gene expression. (**B**) Relative expression levels of IAV-specific genes in A549 cells. Viral RNA levels of M2 and NS genes were quantified by qRT-PCR and normalized to 18S rRNA. CWFs from LP_SD38 exhibited a marked reduction in viral RNA copy numbers compared with LP_WT CWF, indicating a robust and specific interaction mediated by nanobody display. (**C**) Schematic representation of the infection inhibition assay using LP-based nanosomes. IAV particles were pre-incubated with either LP nanosomes or SD38-displaying LP nanosomes (LP_SD38) prior to infection of A549 cells. After infection, total RNA was extracted, and viral replication was quantified by qRT-PCR. (**D**) Relative expression levels of IAV-specific genes in infected A549 cells. Viral RNA levels were normalized to 18S rRNA. Cells infected with IAV pre-treated with LP_SD38 nanosomes showed a significant decrease in M2 and NS gene expression compared with LP nanosome or IAV-alone groups, indicating that surface-displayed SD38 nanobody effectively neutralized viral entry and replication. Data are presented as mean ± SD, and statistical significance was determined by one-way ANOVA with post hoc tests (*p < 0.05, **p < 0.01, ***p < 0.001, ****p < 0.0001; ns, not significant).

For binding assays, IAV particles were pre-incubated with the CWFs and subsequently used to infect A549 human lung epithelial cells. Viral replication was then quantified by measuring IAV-specific gene expression (Fig. 3A). CWFs derived from LP_SD38 markedly reduced the expression levels of both IAV nonstructural (NS) and matrix protein 2 (M2) genes compared with either the LP CWF control group or the IAV-only group (Fig. 3B). To further evaluate the antiviral efficacy of nanobody-displaying minicells themselves, we performed additional infection assays using purified LP nanosomes and LP_SD38 nanosomes (Fig. 3C). Consistent with the CWF-based results, LP_SD38 nanosomes significantly suppressed IAV M2 and NS gene expression in infected A549 cells relative to LP nanosomes or IAV alone (Fig. 3D). These findings provide direct evidence that engineered bacterial membranes and corresponding nanosomes can physically associate with and neutralize IAV particles through nanobody-mediated interactions. Building on this confirmation of viral binding and neutralization, we next investigated whether nanobody-expressing minicells, hereafter referred to as “neutralizing nanosomes,” could exert antiviral activity *in vivo*.

### *In vivo* Neutralization of IAV by LP_SD38 Nanosome

**Fig. 4:**
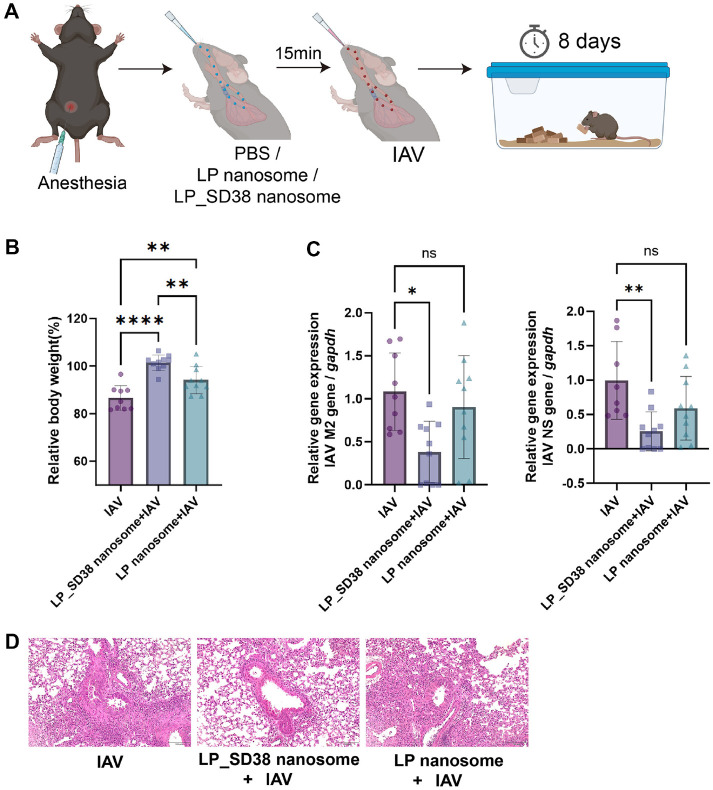
*In vivo* neutralization of IAV by SD38 expressing minicells. (**A**) Experimental design. Mice were anesthetized and intranasally administered with PBS, nanobody-negative minicells (LP nanosome), or nanobody-expressing minicells (LP_SD38 nanosome). After 15 min, mice were challenged with 10^4^ PFU of IAV and monitored for 8 days. (**B**) Relative changes in body weight at 8 days post-infection, expressed as a percentage of initial body weight. Mice treated with LP_SD38 nanosome exhibited significantly greater recovery compared with the IAV-only and LP nanosome groups. (**C**) Relative viral gene expression in lung tissue. Viral RNA levels of M2 and NS genes were measured by qRT-PCR and normalized to *gapdh*. The LP_SD38 nanosome group exhibited a significant reduction in IAV NS and M2 gene expression compared with control groups. (**D**) Representative histopathological images of lung tissues collected at 8 days post-infection. Lung sections from IAV-infected mice exhibited severe peribronchial and perivascular immune cell infiltration, whereas those from mice treated with LP_SD38 nanosomes showed markedly reduced inflammatory cell accumulation and alveolar damage compared with the IAV-only and LP nanosome groups. Scale bar = 100 μm. Data are presented as mean ± SD, and statistical significance was determined by oneway ANOVA with post hoc tests (*p < 0.05, **p < 0.01, ****p < 0.0001; ns, not significant).

To evaluate the protective efficacy of SD38-expressing nanosomes, we established a murine model of IAV infection (Fig. 4A). Mice were anesthetized and intranasally pretreated with PBS, nanobody-negative minicells (LP nanosomes), or SD38-expressing minicells (LP_SD38 nanosomes). After 15 min, animals were inoculated with 10^4^ PFU of IAV, and body weight was monitored for 8 days. Mice treated with LP_SD38 nanosome showed a significant recovery of body weight loss induced by IAV infection, whereas animals receiving LP nanosome exhibited less pronounced weight recovery (Fig. 4B). This indicates that LP_SD38 nanosome provided substantial protection against IAV-induced morbidity. Consistent with these physiological outcomes, qRT-PCR analysis of lung tissue at 8 days post-infection revealed a marked reduction in the expression of the IAV NS and M2 genes in the LP_SD38 nanosome group (Fig. 4C). In contrast, treatment with LP nanosomes did not significantly reduce viral RNA levels compared with the IAV-only group (Fig. 4C). In agreement with these molecular findings, histopathological analysis of lung tissues further supported the protective role of LP_SD38 nanosomes (Fig. 4D). Hematoxylin and eosin–stained sections revealed severe peribronchial and perivascular infiltration of inflammatory cells and alveolar tissue damage in IAV-infected mice. In contrast, lungs from animals pretreated with LP_SD38 nanosomes exhibited markedly reduced inflammation and preserved alveolar structures compared with those from the IAV-only or LP nanosome groups, confirming that SD38-expressing nanosomes effectively mitigated virus-induced pulmonary pathology.

Together, these results demonstrate that intranasal administration of LP_SD38 nanosomes effectively neutralizes IAV *in vivo* by alleviating physiological symptoms and limiting viral replication in the respiratory tract. These findings highlight the potential of minicell-based nanobody delivery as a promising prophylactic or therapeutic strategy against influenza virus infection.

## Discussion

In this study, we report the construction of a recombinant *L. plantarum* system capable of presenting nanobodies on its surface and demonstrate its efficacy in neutralizing IAV both *in vitro* and *in vivo*. Our findings highlight a novel strategy that integrates the safety and probiotic properties of *L. plantarum* with nanobody-display modules, resulting in “neutralizing nanosomes” that directly engage and neutralize viral particles. The successful anchoring of the nanobody constructs to the bacterial cell wall validates the robustness of our plasmid design and surface-display strategy [19]. Importantly, the engineered minicells retained intact surface presentation, as confirmed by immunofluorescence microscopy, and displayed nanobody molecules in a structurally accessible and functional conformation. These features are critical because they allow nanobody-expressing bacteria CWFs to interact directly with IAV particles, as demonstrated by the binding assays using viral RNA quantification. This establishes proof-of-concept that engineered bacterial surfaces can be transformed into living or cell-derived virus-trapping platforms.

The *in vivo* studies further emphasize the translational potential of this approach. Intranasal administration of nanobody-expressing nanosome significantly mitigated IAV-induced weight loss and reduced viral burden in the lungs, underscoring the protective effect of this platform. The ability to achieve viral neutralization through local delivery at the respiratory mucosa is particularly advantageous for combating influenza, given that the virus initiates infection at mucosal surfaces. Unlike conventional vaccines or antiviral drugs, our nanosome system provides an immediate neutralizing barrier against viral particles, potentially bridging the gap between exposure and immune activation. Interestingly, administration of wild-type *L. plantarum* nanosomes also led to a modest but statistically significant improvement in body weight compared with the virus-only group, although the effect was considerably weaker than that observed with SD38-expressing nanosomes. This modest effect may be attributable to nanosome-mediated “adjuvant-like” stimulation of mucosal immunity, as the principal component of the nanosome surface is peptidoglycan, a well-known immune stimulator. This intriguing phenomenon clearly warrants further detailed investigation. From a broader perspective, this study introduces a new class of biologically derived materials for antiviral intervention. Recent advances in vaccine design have leveraged multi-antigen (multi-epitope)–recognizing nanobodies, yielding broad and potent protection [22-24]. In line with these developments, probiotic-derived minicells offer intrinsic safety advantages owing to their chromosome-free nature and can be manufactured at scale using established fermentation workflows. Moreover, the modular architecture of our platform enables rapid swapping of nanobody specificities, allowing swift retargeting to newly emerging influenza variants—or even to unrelated pathogens. Such adaptability is particularly valuable in pandemic settings, where speed of development, manufacturability, and breadth of applicability are paramount.

In conclusion, we present recombinant *L. plantarum*-derived nanobody minicells as a promising and versatile antiviral platform. By combining the stability and safety of bacterial minicells with the specificity of nanobody-mediated neutralization, this system represents a novel material for virus neutralization research and a potential prophylactic or therapeutic strategy against influenza and other viral infections. Future studies will focus on optimizing the breadth of neutralization, scaling production, and exploring applications beyond influenza, further establishing nanobody minicells as a next-generation antiviral modality.

## References

[1] Jain R, Sharma H, Pena L, Jit S, Rathi B, De Oliveira RN (2025). Influenza virus: genomic insights, evolution, and its clinical presentation. Microb. Pathog..

[2] Rossman JS, Lamb RA (2011). Influenza virus assembly and budding. Virology.

[3] Abdelwhab EM, Veits J, Mettenleiter TC (2013). Genetic changes that accompanied shifts of low pathogenic avian influenza viruses toward higher pathogenicity in poultry. Virulence.

[4] Charostad J, Rukerd MRZ, Mahmoudvand S, Bashash D, Hashemi SMA, Nakhaie M (2023). A comprehensive review of highly pathogenic avian influenza (HPAI) H5N1: an imminent threat at doorstep. Travel Med. Infect. Dis..

[5] Kang M, Wang LF, Sun BW, Wan WB, Ji X, Baele G (2024). Zoonotic infections by avian influenza virus: changing global epidemiology, investigation, and control. Lancet Infect. Dis..

[6] Xue KS, Moncla LH, Bedford T, Bloom JD (2018). Within-host evolution of human influenza virus. Trends Microbiol..

[7] Ort JT, Shepard SS, Zolnoski SA, Lam TT, Davis CT, Neher RA (2025). Development of avian influenza A(H5) virus datasets for Nextclade enables rapid and accurate clade assignment. Virus Evol..

[8] World Health Organization. Avian influenza weekly update number 1014. 12 September 2025. Geneva: WHO, 2025.

[9] Mostafa A, Nogales A, Martinez-Sobrido L (2025). Highly pathogenic avian influenza H5N1 in the United States: recent incursions and spillover to cattle. NPJ Viruses.

[10] Hu Z, Ai H, Wang Z, Huang S, Sun H, Xuan X (2025). Impact of inactivated vaccine on transmission and evolution of H9N2 avian influenza virus in chickens. NPJ Vaccines.

[11] Min KD, Yoo DS. Ecological drivers for poultry farms predisposed to highly pathogenic avian influenza virus infection during the initial phase of the six outbreaks between 2010-2021: a nationwide study in South Korea. *Front. Vet. Sci.* **10:** 1278852. 10.3389/fvets.2023.1278852 38130434 PMC10733472

[12] Wu NC, Ellebedy AH (2024). Targeting neuraminidase: the next frontier for broadly protective influenza vaccines. Trends Immunol..

[13] Jin BK, Odongo S, Radwanska M, Magez S (2023). NANOBODIES(R): a review of diagnostic and therapeutic applications. Int. J. Mol. Sci..

[14] Mohammed A, Muustafa MI (2025). Nanobodies: a new frontier in antiviral therapies. SLAS Discov..

[15] Laursen NS, Friesen RHE, Zhu XY, Jongeneelen M, Blokland S, Vermond J (2018). Universal protection against influenza infection by a multidomain antibody to influenza hemagglutinin. Science.

[16] Farley MM, Hu B, Margolin W, Liu J. Minicells, back in fashion. *J. Bacteriol.* **198:** 1186-1195. 10.1128/JB.00901-15 26833418 PMC4859596

[17] Yu H, Khokhlatchev AV, Chew C, Illendula A, Conaway M, Dryden K (2021). Minicells from highly genome reduced *Escherichia coli*: cytoplasmic and surface expression of recombinant proteins and incorporation in the minicells. ACS Synth. Biol..

[18] Park J, Chang S, Kang H, Yi S, Jang IH, Lee KA (2025). A safe and versatile minicell platform derived from *Lactiplantibacillus plantarum* for biotechnological applications. J. Microbiol. Biotechnol..

[19] Jang IH, Jung IR, Suh H, Ahn HJ, Kim D, Choi YS (2025). A nasal vaccine displaying anthrax antigen on the surface of *Lactiplantibacillus plantarum* induces protective mucosal immunity against anthrax toxin. J. Microbiol. Biotechnol..

[20] Kang H, Kim D, Kim J (2025). Protocol for the generation and purification of minicells from *Lactiplantibacillus plantarum*. J. Microbiol..

[21] Kim JI, Lee S, Lee GY, Park S, Bae JY, Heo J (2019). Novel small molecule targeting the hemagglutinin stalk of influenza viruses. J. Virol..

[22] Zhou L, Zhou HL, Wang PY, Xu H, Wu JY, Zhou YZ (2024). Construction of engineered probiotic that adhere and display nanobody to neutralize porcine reproductive and respiratory syndrome virus. Arch. Microbiol..

[23] Xu S, Liu Y, Luo C, Zhou M, Wang K, Xie Q (2025). Identification and characterization of a broadly neutralizing and protective nanobody against the HA1 domain of H5 avian influenza virus hemagglutinin. J. Virol..

[24] Verma V, Sinha N, Raja A (2025). Nanoscale warriors against viral invaders: a comprehensive review of Nanobodies as potential antiviral therapeutics. MAbs.

